# Recalibrating the Village: Nurturing Holistic Well-being for Black Older Adults

**DOI:** 10.1093/geroni/igaf122.3248

**Published:** 2025-12-31

**Authors:** Raymond A Jetson

**Affiliations:** Aging While Black, Baton Rouge, Louisiana, United States

## Abstract

In the face of persistent disparities and social inequities, Black older adults continue to demonstrate remarkable fortitude, often rooted in the strength of their communities – the modern-day “villages” that provide multifaceted support. We explore how these existing community structures can be leveraged and enhanced to promote comprehensive well-being among Black older adults, addressing the complex interplay of factors that contribute to quality of life in later years. Drawing on recent studies and innovative community initiatives, we will examine how traditional support systems like churches, fraternal organizations, and community centers are evolving to meet the multidimensional needs of today’s Black older adults. Through case studies of programs that have successfully “recalibrated” traditional community structures to address contemporary challenges holistically, we will describe church-based wellness initiatives that integrate physical, mental, and spiritual health; fraternity-led mentorship programs that foster purpose and social engagement for older men; and community center-based initiatives that address digital empowerment, financial wellness, and creative expression. Our presentation will conclude with a framework for gerontologists, policymakers, and community leaders to assess and enhance the “village” support system in their own communities. This framework emphasizes culturally relevant approaches that honor the strength and determination of Black older adults while addressing their unique experiences and needs. By recalibrating these vital community networks through a holistic lens, we can create more robust, responsive, and effective support systems for Black older adults, ultimately improving their overall well-being, sense of belonging, and quality of life across multiple dimensions.

